# Supplementary material for: Microbiome analysis of Pacific white shrimp gut and rearing water from Malaysia and Vietnam: implications for aquaculture research and management
Source: PeerJ. 2018 Oct 30;6:e5826. doi: 10.7717/peerj.5826 (PMC6214229; doi:10.7717/peerj.5826)
Supplement: Table S3 [file peerj-06-5826-s003.docx]

Supplementary Table 2: Top 3 Blast hits of the five most abundance chloroplast-derived ASVs.

| ASV | GenBank ID | Identity | Description | Common Name |
| --- | --- | --- | --- | --- |
| ASV12 | FN563101.1 | 98.762 | Pyramimonas disomata M1802 | green algae |
|  | FN563100.1 | 96.287 | Cymbomonas tetramitiformis M1669 | green algae |
|  | KX013545.1 | 96.287 | Cymbomonas tetramitiformis strain PLY262 | green algae |
| ASV21 | JN207220.1 | 99.507 | Virgulinella fragilis clone W613-140 | diatom |
|  | JN207219.1 | 99.261 | Virgulinella fragilis clone N815-11 | diatom |
|  | KJ958479.1 | 99.015 | Chaetoceros simplex | diatom |
| ASV143 | FJ002215.1 | 99.506 | Chaetoceros calcitrans isolate C07 | diatom |
|  | JN207220.1 | 99.259 | Virgulinella fragilis clone W613-140 | diatom |
|  | JN207230.1 | 99.012 | Virgulinella fragilis clone W622-9 | diatom |
| ASV149 | KJ958481.1 | 99.754 | Cyclotella sp. WC03_2 | diatom |
|  | FJ002189.1 | 99.754 | Cyclotella cryptica isolate C114 | diatom |
|  | KJ958480.1 | 99.509 | Cyclotella sp. L04_2 | diatom |
| ASV156 | AY702148.1 | 98.429 | Picochlorum sp. RCC289 | green algae |
|  | AY702138.1 | 98.429 | Trebouxiophyceae sp. DJS-2004 | green algae |
|  | AY702135.1 | 98.429 | Nannochloris sp. 2-RCC13 | green algae |
