# Supplementary material for: Microbiome analysis of Pacific white shrimp gut and rearing water from Malaysia and Vietnam: implications for aquaculture research and management
Source: PeerJ. 2018 Oct 30;6:e5826. doi: 10.7717/peerj.5826 (PMC6214229; doi:10.7717/peerj.5826)
Supplement: Table S4 [file peerj-06-5826-s004.docx]

Supplementary Table 4: Summary of top three most abundant phyla in previous microbiome studies.

| **References** | **Top 3 most abundant phyla** | | | | | |
| --- | --- | --- | --- | --- | --- | --- |
|  | **Pond water** | | | **Shrimp instestines** | | |
| Tang et al., 2014 | Actinobacteria (53.5%) | Proteobacteria (18.8%) | Bacteroidetes (4.3%) |  |  |  |
| Zhang et al., 2014 | Cyanobacteria (0.7%) | Bacteroidetes (0.2%) | Actinobacteria (0.04%) | Proteobacteria (57.0%) | Tenericutes (41.4%) | Firmicutes (1.1%) |
| Xiong et al., 2015 | Proteobacteria | Bacteroidetes | Actinobacteria | Proteobacteria | Plancytomycetes | Bacteroidetes |
| Hou et al., 2016 | Actinobacteria | Proteobacteria | Cyanobacteria |  |  |  |
| Huang et al., 2016 | Proteobacteria | Actinobacteria | Bacteroidetes | Proteobacteria | Bacteroidetes | Actinobacteria |
| Rungrassamee et al., 2016 | Proteobacteria (54.1%) | Actinobacteria (27.5%) | Deinococcus-Thermus (15.2%) | Proteobacteria (82.2%) | Firmicutes (10.7%) | Bacteroidetes (5.2%) |
| Zhang et al., 2016 | Proteobacteria (43.4%) | Actinobacteria (36.8%) | Cyanobacteria/Chloroplast (14.3%) |  |  |  |
| Cornejo-Granados et al., 2017 |  |  |  | Proteobacteria (69.3%) | Cyanobacteria (24.3%) | Tenericutes (1.5%) |
| Xiong et al. 2017 |  |  |  | Proteobacteria (67.8%) | Bacteroidetes (14.1%) | Actinobacteria (9.2%) |
| Zeng et al., 2017 |  |  |  | Proteobacteria (63.5%) | Cyanobacteria (7.0%) | Tenericutes (6.5%) |
| This study - Malaysian samples | Actinobacteria (31.1%) | Cyanobacteria (27.1%) | Proteobacteria (19.1%) | Proteobacteria (60.5%) | Fusobacteria (15.9%) | Cyanobacteria (12.3%) |
| This study -Vietnamese samples | Proteobacteria (43.5%) | Bacteroidetes (29.1%) | Actinobacteria (12.3%) | Proteobacteria (74.8%) | Planctomycetes (8.0%) | Bacteroidetes (5.6%) |
